# Cassava brown streak virus evolves with a nucleotide-substitution rate that is typical for the family *Potyviridae*

**DOI:** 10.1016/j.virusres.2024.199397

**Published:** 2024-05-22

**Authors:** Willard Mbewe, Settumba Mukasa, Mildred Ochwo-Ssemakula, Peter Sseruwagi, Fred Tairo, Joseph Ndunguru, Siobain Duffy

**Affiliations:** a Department of Biological Sciences, Malawi University of Science and Technology, P. O. Box 5196, Limbe, Malawi; b School of Agriculture and Environmental Science, Department of Agricultural Production, P. O. Box 7062, Makerere University, Kampala, Uganda; c Mikocheni Agricultural Research Institute, P.O. Box 6226, Dar es Slaam, Tanzania; d Department of Ecology, Evolution and Natural Resources, Rutgers University, New Brunswick, NJ 08901, United States

**Keywords:** Cassava brown streak virus, Evolution, Substitution rate, Metaanalysis

## Abstract

The ipomoviruses (family *Potyviridae*) that cause cassava brown streak disease (cassava brown streak virus [CBSV] and Uganda cassava brown streak virus [UCBSV]) are damaging plant pathogens that affect the sustainability of cassava production in East and Central Africa. However, little is known about the rate at which the viruses evolve and when they emerged in Africa – which inform how easily these viruses can host shift and resist RNAi approaches for control. We present here the rates of evolution determined from the coat protein gene (CP) of CBSV (Temporal signal in a UCBSV dataset was not sufficient for comparable analysis). Our BEAST analysis estimated the CBSV CP evolves at a mean rate of 1.43 × 10^−3^ nucleotide substitutions per site per year, with the most recent common ancestor of sampled CBSV isolates existing in 1944 (95% HPD, between years 1922 – 1963). We compared the published measured and estimated rates of evolution of CPs from ten families of plant viruses and showed that CBSV is an average-evolving potyvirid, but that members of *Potyviridae* evolve more quickly than members of *Virgaviridae* and the single representatives of *Betaflexiviridae, Bunyaviridae, Caulimoviridae* and *Closteroviridae.*

## Highlights

• Cassava brown streak virus’ CP evolves at a similar rate to other potyvirid CPs.
• CBSV originated between 1923 and 1963, consistent with CBSD's description in the 1930s.
• Members of *Potyviridae* evolve faster than those in five other plant virus families.

## 1 Introduction

Cassava brown streak virus (CBSV) and Ugandan cassava brown streak virus (UCBSV) are the two viruses implicated in etiology of cassava brown streak disease (CBSD) (Monger et al., 2001; Mbanzibwa et al., 2009; Winter et al., 2010), a threat to food security to more than 300 million people in Africa (Patil et al., 2015; Robson et al., 2023). These viruses belong to the genus *Ipomovirus* and family *Potyviridae* (Inoue-Nagata et al., 2022). These viruses are transmitted by whiteflies (most often *Bemisia tabaci* (Gennadius) (Maruthi et al., 2005) and disseminated by planting of infected cuttings.

CBSVs, like other members of the genus *Ipomovirus,* have a positive-sense single strand RNA (+ssRNA) genome that is translated into a polyprotein precursor that is cleaved by viral proteases to produce ten mature proteins denoted as P1, P3, 6K1, CI, 6K2, VPg, NIa-Pro, NIb, HAM1, and CP (Dombrovsky et al., 2014). An additional peptide, PIPO, is translated in the +2 reading frame relative to P3 via ribosomal frameshifting or transcriptional slippage at a high conserved motif at the 5′ end of PIPO (Chung et al., 2008).

The functions of CP gene of in members of *Potyviridae* (which we will informally refer to as ‘potyviruses’ – not just the members of genus *Potyvirus*) have been well documented (Atreya et al., 1990; Rybicki and Shukla, 1992; Varrelmann and Maiss, 2000; Dombrovsky et al., 2014). The CP is generally associated with the success of viral infection: it plays a role in vector transmission virion assembly, stability, and systematic infection, making it one of the most important genes for virus pathogenicity (Atreya et al., 1990; Varrelmann and Maiss, 2000). Thus, it is not surprising that the CP gene is frequently used to resolve phylogenetic relationships among viral isolates (Abdalla and Ali, 2021).

Although mechanisms driving faster evolutionary rates in CBSV than UCBSV were determined (Alicai et al., 2016), there is still limited knowledge on the rate of molecular evolution and the age of the sampled genetic diversity, reflected in the time to the most recent common ancestor (TMRCA). This information is critical to understanding the evolutionary fingerprint of any plant virus (Kosakovsky et al., 2010), and particularly whether they exhibit reduced rates of evolutionary change, which in turn may have major implications on their ability to emerge in new host species or evade RNAi approaches for control (Prins et al., 2008; Patil et al., 2011). We undertook this study to estimate the nucleotide substitution rate of CBSV and UCBSV and calculate how recently these viruses may have emerged in cassava by the time to most recent common ancestor (TMRCA). We used CP sequences because there were more sequences of this genomic region, with reliable dates of isolation, over a wider date range, than of other genes or whole genomes in GenBank. The isolates used here in were collected in East and Central Africa from 1996 to 2019: the period from when CBSV was first demonstrated to cause CBSD (Monger et al., 2001) to date. We then compared the rate of evolution of CBSV to those previously reported for CPs of other plant viruses.

## 2 Materials and methods

### 2.1 Nucleotide sequences and alignment

Nucleotide sequences of full-length CP gene of CBSVs with known dates of isolation were downloaded from GenBank on 3rd September 2016. These sequences were from Kenya, Tanzania and Mozambique. Alignments were performed by ClustalW (Larkin et al., 2007) and manually edited using Se-Al (http://tree.bio.ed.ac.uk/software). Sequences were screened for recombination using six recombination detection programs within the RDP4 package (http://darwin.uvigo.es/rdp/rdp/html): RPD, GENCONV, MaxChi, Chimaera, Bootscan and 3Seq (Martin et al., 2015). The default detection thresholds were employed in all cases except that sequences were ‘set linear’. The Bonferroni correction cutoff was set at highest acceptable P-value of 0.05. Sequences were considered recombinant when identified by at least three of the algorithms, and recombinant sequences were removed from the gene alignments. This ensured that the results have a high likelihood of being unaffected by recombination.

### 2.2 Molecular clock signal analysis

Maximum-Likelihood (ML) tree topologies were constructed from the alignments with the use of the best fitting nucleotide substitution model, the General Time Reversible with invariant sites and a gamma distribution (GTR+*I*+Γ_4_, Tamura et al., 2021). We deployed an interactive regression approach to explore the association between CBSVs’ CP genetic divergence through time and sampling dates by running TempEst (Rambaut et al., 2016). This allowed an evaluation of the temporal signal and assessment of how well a strict molecular clock might fit each of the data sets, as well allowed identification of potential outliers that deviated from the standard regression.

### 2.3 Estimation of TMRCA and epidemic reconstruction

The CBSV dataset was prepared for Bayesian MCMC analysis in BEAUti v 2.4.3, and then run in BEAST v1.10.4 (Suchard et al., 2018). We used marginal likelihoods via stepping stone sampling for three molecular clock models (strict, relaxed lognormal, and random local) and three demographic models (constant, exponential and Bayesian skyline) (Baele et al., 2016). After these 9 models were run for 100 million steps and evaluated, the best fitting model was run for 300 million steps (ensuring all parameters reached an ESS value ≥200). A control without sequence data was also run to ensure the priors did not independently determine the results of the analysis. Uncertainty in parameter estimates were reflected in the 95% highest probability density (HPD) values. Convergence in each BEAST run was assessed in Tracer v 1.6.0 (http://tree.bio.ed.ac.uk/software/tracer). The log and tree file for the relaxed lognormal clock with Bayesian skyline population priors were used for the reconstruction of the viral effective population size (Heled and Drummond, 2008, 2010; Parag et al., 2022). As further checks of the temporal signal in the dataset, we ran an isochronous version of the dataset (sequences identical, but all dated to the same year) with the best fitting model priors, and calculated the log Bayes Factor to determine if the tip-dated dataset was a better fit than the isochronous dataset (Duchene et al., 2020), and a Mantel test was run (using R scripts from Murray et al. 2016) to examine the degree of confounding between sampling date and phylogenetic clustering.

### 2.4 Selection analysis

Processes of natural selection over time mold every gene into a mosaic of sites that evolve rapidly or resist change, often captured in the site-specific ratio of non-synonymous to synonymous substitution rates (dN/dS, Kosakovsky et al., 2010). We deployed per-codon analyses using the following codon-based ML algorithms implemented in the web-server DATAMONKEY (http://www.datamonkey.org) (Kosakovsky and Frost, 2005): single-likelihood ancestor counting (SLAC) method, internal fixed method effects method (IFEL), Fixed Effects Likelihood (FEL) (Kosakovsky and Frost 2005; Massingham and Goldman, 2005) and the recently described Mixed Effects Model of Evolution (MEME) and Fast Unbiased Bayesian Approximation (FUBAR) (Murrell et al., 2013). All these methods incorporated the GTR model of nucleotide substitution, with phylogenetic trees inferred using the neighbor-joining method.

### 2.5 Comparison with other plant virus CP substitution rates

A survey of the literature revealed 42 published estimated substitution rates of plant virus coat/capsid proteins, mostly obtained with similar methods as in this study (i.e., BEAST analysis). All averages were calculated on log-transformed values. Statistical analyses and plotting were done in MS Excel.

## 3 Results

### 3.1 Temporal signal for UCBSV and CBSV

TempEst's molecular clock analysis of CBSV and UCBSV data sets placed the estimated TMRCA, based on crude root-to-tip regressions, at 1983 and 1575, respectively. The R^2^ values were 0.23 for CBSV and 0.06 for UCBSV (Supplementary Figure 1a and 1b). Thus, the temporal signal of the UCBSV dataset was not well supported and consequently no further analyses were conducted with the UCBSV dataset. The final CBSV alignment, which did show temporal signal, is available as supplementary file S1.

### 3.2 Evolutionary history of CBSV

**Fig. 1:**
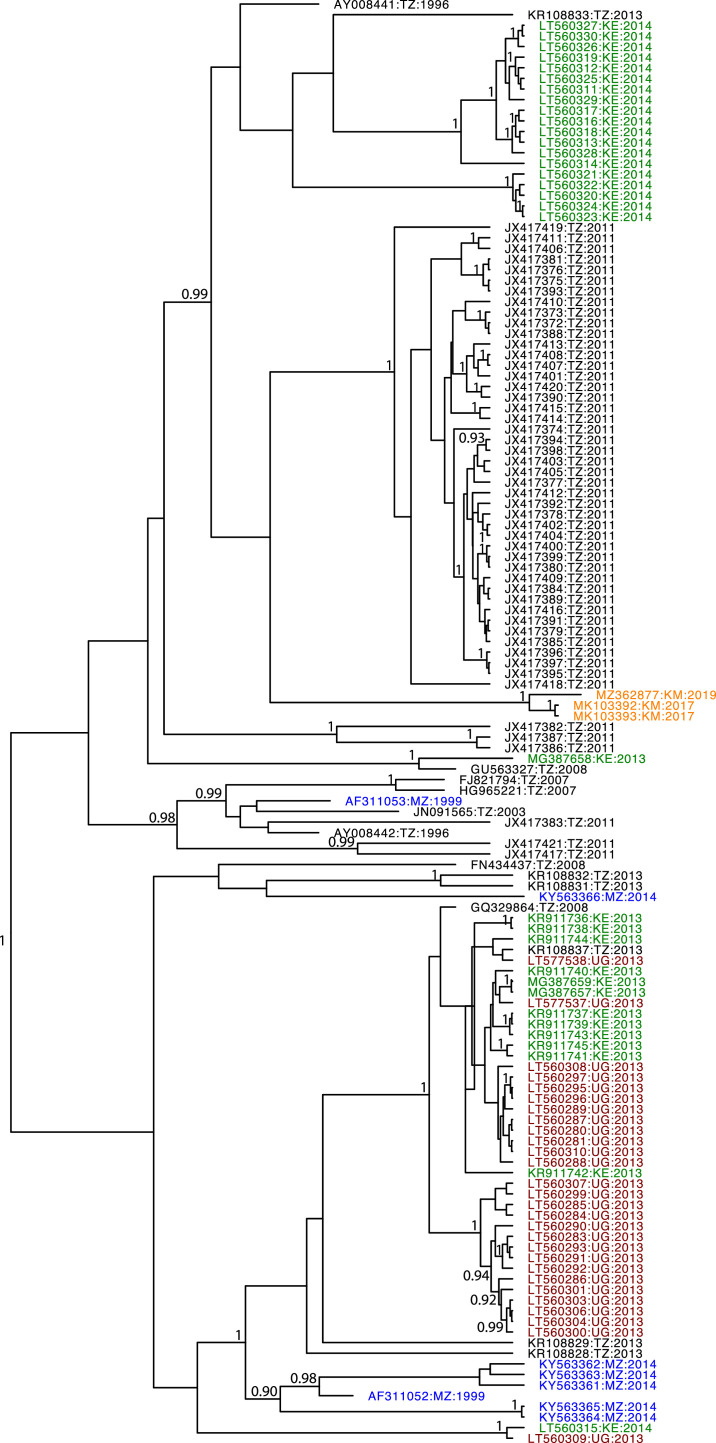
The Maximum Clade Credibility (MCC) tree for the CBSV CP data set, based on a 300 million step run with a relaxed lognormal molecular clock and a Bayesian skyline demographic prior. Taxa are plotted by year of isolation. Posterior probability values above 0.9 are shown. Taxa are colored by country of isolation. The majority of the sequences are from Tanzania, and are shown in black, Kenyan isolates are shown in green, Ugandan isolates in brown, isolates from Mozambican isolates are shown in blue, and isolates from Comoros are shown in orange.

**Fig. 2:**
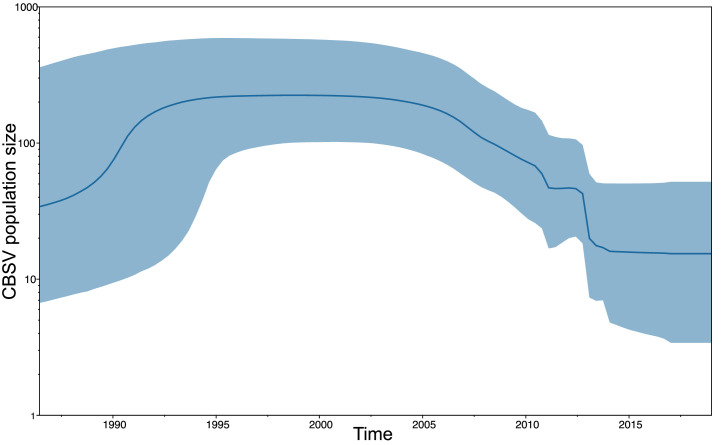
Bayesian reconstruction of the effective population size of CBSV showing viral population dynamics over time. Sequences used in the tip-dated analysis were sampled from 1996 to 2019.

All 136 CP gene sequences of CBSV were included in the analyses as no recombination within the CP gene was detected. Bayesian coalescent methods were used to determine the rate of evolution and TMRCA were estimated using from this dataset of CBSV-CP gene sequences obtained over a period of 24 years (1996 to 2019). The TempEst results did not produce a very strong correlation between genetic divergence (*r* = 0.48) and time, and stepping stone sampling indicated that a relaxed molecular clock with a Bayesian Skyline demographic prior was the most appropriate model to use (MLE = −5605.34), which was a similar to that for the random local clock with Bayesian skyline demographic prior (MLE = −5605.86); other combinations of clock and demographic models were between −5615.06 and −5628.87). This tip-dated, heterochronous analysis was a much better fit to the data than a control analysis that examined the data as if all 136 sequences were isolated in the same year (log Bayes Factor = 349), indicating substantial temporal signal in our CBSV dataset. The mean rate of nucleotide substitution for relaxed log normal clock and Bayesian skyline prior is 1.43 × 10^−3^ nucleotide substitutions/site/year (n/s/y, 95% HPD, 1.4 × 10^−4^ – 8.86 × 10^−3^). The TMRCA for the CBSV dataset was the year 1944 (95% HPD, between years 1922 to 1963). The maximum clade credibility tree is shown in Fig. 1. The population change over time is visualized in a Bayesian skyline plot (Fig. 2). It shows that, despite epidemic spread in recent decades, the diversity of CBSV sequences declined between 2005 and 2015.

**Fig. 3:**
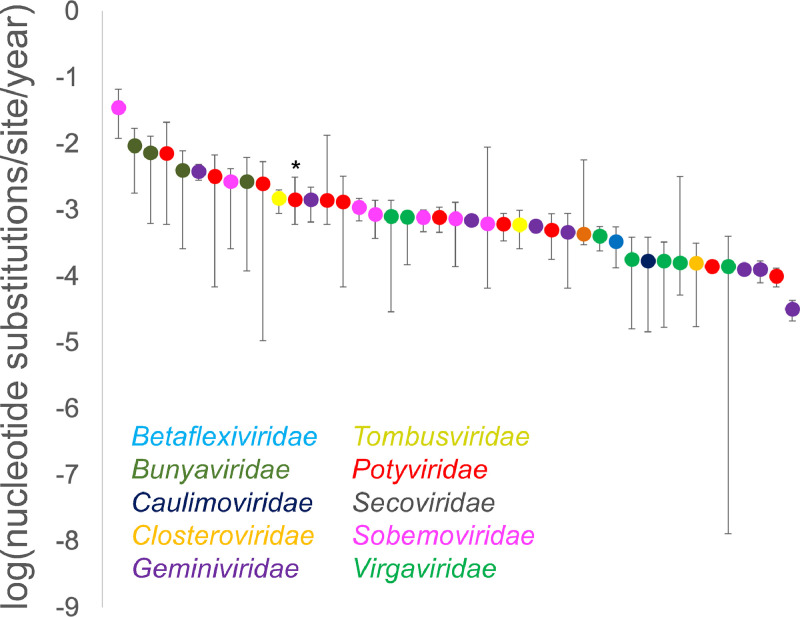
Rates of evolution of plant virus CP genes. Error bars reflect 95% HPDs, and estimates without error bars were calculated without BEAST (Table 1). The rate for CBSV is indicated with an asterisk.

To further assess the temporal signal in our CBSV dataset, we conducted a Bayesian estimate of temporal signal, comparing our tip-dated estimate with one where the same sequences are considered to have been sampled in the same year. The comparison between the heterochronous and isochronous analyses strongly supported temporal signal in the data set (log Bayes Factor = 349). However, it appears that a CBSV isolate's location in the phylogenetic tree (Fig. 1) is correlated with its date of isolation, as is common in studies of viruses, especially during outbreaks (Murray et al. 2016). A Mantel test showed relatively a statistically significant, but fairly low correlation between genetic clustering and date of isolation in our analysis (*r* = 0.25, *p* = 0.001). Ten replicates with a clustered permutation test showed that the majority of replicates produced similar substitution rates and TMRCAs (supplemental file 2) – consistent with confounding between sampling date and genetic sequence. This reduces confidence in our estimated substitution rate.

#### 3.2.1 Selection pressure

There was weak support for positive section acting upon the CP of CBSV using either SLAC, IFEL, FEL, MEME and FUBAR, with most of the gene under purifying selection. The 18th and 115th codons in the alignment (the 114th and 211st codon of the complete CP gene, relative to the CP of the reference sequence NC_012698.2) were detectably under positive selection by SLAC, FEL and IFEL, FUBAR (BF ≥0.6) and MEME (BF= 0.1).

#### 3.2.2 Substitution rate

The estimated CBSV CP substitution rates are within the range of those previously reported in other RNA and ssDNA plant viruses (Duffy and Holmes, 2008, 2009; Gibbs et al., 2008; Pagán et al., 2010; Pagán and Holmes, 2010; Simmons et al., 2008), and CBSV is in the middle of the rates estimated for other potyviruses (Fig. 1, Table 1). Importantly, none of these other estimated substitution rates were conducted on datasets evaluated as rigorously for temporal signal (e.g., none had conducted a BETS analysis or a clustered permutation date randomization test), and so many may be similarly confounded by clustered isolates having been sampled in the same year. Based on our literature review, potyviruses (family *Potyviridae*) are the most well-studied in terms of evolutionary rates, with 11 different viruses having estimated substitution rates for their CP gene (means ranging from 7.2 × 10^−3^ to 1 × 10^−4^ n/s/y). Three other plant virus families have at least five published substitution rates for different viruses: geminivirus mean rates ranged from 3.8 × 10–3 to 1.3 × 10^−4^ n/s/y, sobemovirus (family *Sobemoviridae*) mean rates ranged from 3.5 × 10–2 to 6.2 × 10^−4^ n/s/y and tobamovirus (family *Virgaviridae*) mean rates were more narrowly distributed from 7.8 × 10^−4^ to 1.4 × 10^−4^ n/s/y.

**Table 1:** Details of evolutionary rates of coat protein gene of various plant viruses.

Virus	Genus (Family)	Date range	Clock model	Growth model	Mean substitution rate		95% HPD substitution rate		Reference
ACMV	*Begomovirus* *(Geminiviridae)*	1928 – 2014	Relaxed	Bayesian Skyline		1.3 × 10^−4^	8.0 × 10^−5^ – 1.7 × 10^−4^	Rieux et al., 2021	
ArMV	*Nepovirus* (*Secoviridae*)	1991 – 2008	Relaxed	Constant		9.3 × 10^−3^	1.8 × 10^−3^ – 1.7 × 10^−2^	Thompson et al., 2014	
BChV	*Polerovirus* *(Solemoviridae)*	1985 – 2005	Relaxed	Bayesian Skyline		2.7 × 10^−3^	2.6 × 10^−4^ – 4.2 × 10^−3^	Pagán and Holmes, 2010	
BYDV	*Luteovirus* *(Tombusviridae)*	1917 – 2008	Relaxed	Bayesian Skyline		1.5 × 10^−3^	9.8 × 10^−4^ – 2.0 × 10^−3^	Pagán and Holmes, 2010	
CABYV	*Polerovirus* *(Solemoviridae)*	2003 – 2008	Relaxed	Bayesian Skyline		3.5 × 10^−2^	1.2 × 10^−2^ – 6.6 × 10^−2^	Pagán and Holmes, 2010	
CABMV	*Potyvirus* *(Potyviridae)*		Relaxed			1.4 × 10^−4^		Gibbs et al., 2008	
CaMV	*Caulimovirus* *(Caulimoviridae)*	1960 – 2010	Relaxed	Exponential		1.71 × 10^−4^	1.45 × 10^−5^ – 3.87 × 10^−4^	Yasaka et al., 2014	
CBSV	*Ipomovirus* *(Potyviridae)*	1996 – 2019	Relaxed	Bayesian Skyline		1.43 × 10^−3^	1.4 × 10^−4^ – 8.86 × 10^−3^	This study	
CGMMV	*Tobamovirus* *(Virgaviridae)*	1970 – 2008	Relaxed	Bayesian Skyline		8.0 × 10^−4^	2.9 × 10^−5^ – 1.4 × 10^−3^	Pagán et al., 2010	
CTV	*Closterovirus* *(Closteroviridae)*	1990 – 2010	Strict	Bayesian Skyline		1.6 × 10^−4^	1.7 × 10^−5^ – 3.2 × 10^−4^	Silva et al., 2012	
CYDV	*Polerovirus* *(Solemoviridae)*	1925 – 2005	Relaxed	Bayesian Skyline		7.4 × 10^−4^	1.4 × 10^−4^ – 1.3 × 10^−3^	Pagán and Holmes, 2010	
CyEVA	*Potyvirus* *(Potyviridae)*	2008 – 2014	Relaxed	Constant		7.15 × 10^−3^	6.11 × 10^−4^ – 2.12 × 10^−2^	Ohshima et al., 2016	
DSV	*Mastrevirus* *(Geminiviridae)*					1.27 × 10^−4^		Ortega-del Campo et al., 2021	
EACMV	*Begomovirus* *(Geminiviridae)*	1996 – 2009	Relaxed			3.83 × 10^−3^	2.81 × 10^−3^ – 4.89 × 10^−3^	De Bruyn et al., 2012	
GFLV	*Nepovirus* (*Secoviridae*)	1991 – 2011	Relaxed	Exponential		7.3 × 10^−3^	6.3 × 10^−4^ – 1.3 × 10^−2^	Thompson et al., 2014	
MSV	*Mastrevirus (Geminiviridae)*	1979 – 2006	Relaxed	Constant		7.0 × 10^−4^	6.2 × 10^−4^ – 7.3 × 10^−4^	Harkins et al., 2009	
GRBV	*Grablovirus (Geminiviridae)*	2010–2017	Strict	Exponential		3.2 × 10^−5^	2.1 × 10^−5^ – 4.3 × 10^−5^	Thompson, 2022	
NDV	*Potyvirus* *(Potyviridae)*	2000 – 2011	Relaxed	Exponential		6.16 × 10^−3^	1.31 × 10^−3^ – 1.34 × 10^−2^	Ohshima et al., 2016	
NLSYV	*Potyvirus* *(Potyviridae)*	2000 – 2013	Relaxed	Constant		1.33 × 10^−3^	6.92 × 10^−5^ – 3.23 × 10^−3^	Ohshima et al., 2016	
NLV	*Potyvirus* *(Potyviridae)*	2004 – 2012	Relaxed	Constant		2.48 × 10^−3^	1.06 × 10^−5^ – 5.35 × 10^−3^	Ohshima et al., 2016	
NYSV	*Potyvirus* *(Potyviridae)*	2004 – 2013	Relaxed	Constant		3.23 × 10–3	1.35 × 10^−3^ – 6.78 × 10^−3^	Ohshima et al., 2016	
ORSV	*Tobamovirus* *(Virgaviridae)*	1960 – 2004	Relaxed	Bayesian Skyline		7.8 × 10^−4^	1.5 × 10^−4^ – 9.2 × 10^−4^	Pagán et al., 2010	
PaLCD	*Begomovirus* *(Geminiviridae)*	1997–2018	Strict	Constant		1.43 × 10^−3^	6.59 × 10^−4^ – 2.22 × 10^−3^	Srivastava et al., 2022	
PLRV	*Polerovirus* *(Solemoviridae)*	1974 – 2008	Relaxed	Bayesian Skyline		6.2 × 10^−4^	6.6 × 10^−5^ – 8.9 × 10^−3^	Pagán and Holmes, 2010	
PMMoV	*Tobamovirus* *(Virgaviridae)*	1972 – 2008	Relaxed	Bayesian Skyline		1.8 × 10^−4^	1.6 × 10^−5^ – 3.9 × 10^−4^	Pagán et al., 2010	
PRSV	*Potyvirus* *(Potyviridae)*	1990 – 2016	Relaxed	Bayesian Skyline		7.7 × 10^−4^	4.7 × 10^−4^ – 5.6 × 10^−4^	Cabrera Mederos et al., 2019	
PVS	*Carlavirus* *(Betaflexiviridae)*	1985 – 2014	Relaxed	Constant		3.3 × 10^−4^	1.3 × 10^−4^ – 5.6 × 10^−4^	Duan et al., 2018	
PVY	*Potyvirus* *(Potyviridae)*	1938 – 2013	Relaxed	Constant		1.0 × 10^−4^	6.9 × 10^−5^ – 1.3 × 10^−4^	Adrian J. Gibbs et al., 2017	
RMV	*Tobamovirus* *(Virgaviridae)*	1950 – 2001	Relaxed	Bayesian Skyline		1.4 × 10^−4^	1.3 × 10^−8^ – 4.0 × 10^−4^	Pagán et al., 2010	
RSV	*Tenuivirus* *(Bunyaviridae)*	1997 – 2013	Strict	Constant		4.3 × 10^−4^	3.0 × 10^−4^ – 5.7 × 10^−3^	He et al., 2017	
RTSV	*Waikavirus (Secoviridae)*	1995 – 2009	Relaxed	Exponential		4.0 × 10^−3^	2.6 × 10^−4^ – 7.8 × 10^−3^	Thompson et al., 2014	
RYMV	*Sobemovirus (Solemovirdae)*	1975 – 2005	Relaxed	Bayesian Skyline		7.7 × 10^−4^	4.7 × 10^−4^ – 1.0 × 10^−3^	Fargette et al., 2008	
SbDV	*Luteovirus* *(Tombusviridae)*	1990 – 2007	Relaxed	Bayesian Skyline		6.0 × 10^−4^	2.6 × 10^−4^ – 9.9 × 10^−4^	Pagán and Holmes, 2010	
ScYLV	*Polerovirus* *(Solemoviridae)*	1989 – 2008	Relaxed	Bayesian Skyline		1.1 × 10^−3^	6.8 × 10^−4^ – 1.5 × 10^−3^	Pagán and Holmes, 2010	
TMGMV	*Tobamovirus* *(Virgaviridae)*	1907 – 2008	Relaxed	Bayesian Skyline		1.7 × 10^−4^	1.7 × 10^−5^ – 3.3 × 10^−4^	Pagán et al., 2010	
TMV	*Tobamovirus* *(Virgaviridae)*	1899 – 2008	Relaxed	Bayesian Skyline		1.6 × 10^−4^	5.2 × 10^−5^ – 3.2 × 10^−3^	Pagán et al., 2010	
ToMV	*Tobamovirus* *(Virgaviridae)*	1975 – 2018	Strict	MASCOT		4.0 × 10^−4^	2.4 × 10^−4^ – 5.6 × 10^−4^	Xu et al., 2021	
ToRSV	*Nepovirus* (*Secoviridae*)	1991 – 2007	Relaxed	Exponential		2.7 × 10^−3^	1.2 × 10^−4^ – 6.2 × 10^−3^	Thompson et al., 2014	
ToSRV	*Begomovirus* *(Geminiviridae)*					5.70 × 10^−4^		Pinto et al., 2021	
TuMV	*Potyvirus* *(Potyviridae)*	1968 – 2007	Relaxed	Exponential		6.1 × 10^−4^	3.4 × 10^−4^ – 8.9 × 10^−4^	Nguyen et al., 2013	
TuYV	*Polerovirus* *(Solemoviridae)*	1980 – 2006	Relaxed	Bayesian Skyline		8.6 × 10^−4^	3.7 × 10^−4^ – 1.4 × 10^−3^	Pagán and Holmes, 2010	
TYLCV	*Begomovirus* *(Geminiviridae)*	1988 – 2006	Relaxed	Exponential		4.6 × 10^−4^	6.6 × 10^−5^ – 8.9 × 10^−4^	Duffy and Holmes, 2008	
ZYMV	*Potyvirus* *(Potyviridae)*	1984 – 2006	Relaxed	Exponential		5.0 × 10–4	1.8 × 10–4 – 8.8 × 10–4	Simmons et al., 2008	

When analyses were conducted with BEAST, the sampling time frame, molecular clock model and demographic prior are listed. Viruses in the table: African cassava mosaic virus (ACMV) arabis mosaic virus (ArMV), beet chlorosis virus (BChV), barley yellow dwarf virus (BYDV), cucurbit aphid-borne yellows virus (CABYV), cowpea aphid-borne mosaic virus (CABMV), cauliflower mosaic virus (CaMV), cassava brown streak virus (CBSV), citrus tristeza virus (CTV), cucumber green mottle mosaic virus (CGMMV), cyrtanthus elayus virus A (CyEVA), cereal yellow dwarf virus (CYDV), digitaria streak virus (DSV), East African cassava mosaic virus (EACMV), grapevine fanleaf virus (GFLV), grapevine red blotch virus (GRBV), maize streak virus (MSV), narcissus degeneration virus (NDV), narcissus latent virus (NLV), narcissus late season yellows virus (NLSYV), narcissus yellow stripe virus (NYSV), odontoglossum ringspot virus (ORSV), papaya leaf curl disease-causing viruses (PaLCD), pepper mild mottle virus (PMMoV), potato leaf roll virus (PLRV), papaya ringspot virus (PRSV), potato virus S (PVS), potato virus Y (PVY), ribgrass mosaic virus (RMV), rice stunt virus (RSV), rice tungro spherical virus (RTSV), rice yellow mottle virus (RYMV), soybean dwarf virus (SbDV), sugarcane yellow leaf virus (ScYLV), tobacco mild green mosaic virus (TMGMV), tobacco mosaic virus (TMV), tomato mosaic virus (ToMV), tomato ringspot virus (ToRSV), tomato severe rugose virus (ToSRV), turnip mosaic virus (TuMV), turnip yellows virus (TuYV), tomato yellow leaf curl virus (TYLCV), zucchini yellow mosaic virus (ZYMV).

**Fig. 4:**
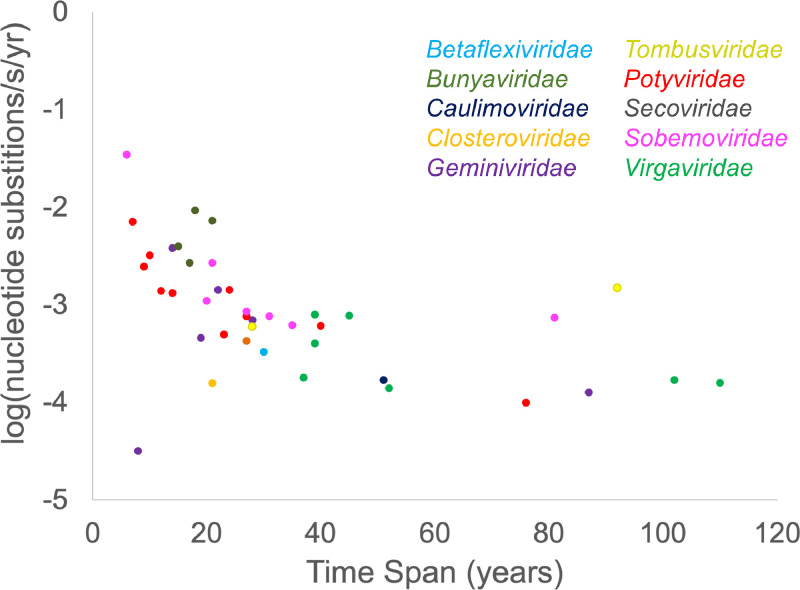
The subset of CP substitutions rates calculated with BEAST are plotted against the time span of sampled viral sequences. The ten viral families are color-coded as in Fig. 3.

Some of the variation in substitution rates is due to the different lengths of time sequences were sampled in these different analyses. Shorter time spans are known to inflate estimated substitution rates (Harrison et al., 1997; Ho et al., 2005) and in fact some published rates of evolution for CP genes of plant viruses in the literature are based on datasets so short that they are acknowledged to be unreliable, and were therefore excluded from our metanalysis (e.g., blackcurrent reversion virus and strawberry mottle virus (Thompson et al., 2014)). We still see that signature of shorter sampling times affecting some of the rates included in our literature survey, with the noticeable outlier of a low rate for GRBV calculated from a dataset of only 8 years (Fig. 4). This unusually low rate is from a multiple-species analysis, looking at the diversification of grabloviruses over a much longer time frame than the other BEAST analyses included here, and therefore isn't perfectly comparable to studies looking at just the most recent common ancestor of a single species (Thompson 2022). The unequal sampling times also makes it more difficult to compare across studies. For instance, potyviruses in this dataset evolve more quickly than tobamoviruses (one-tailed *t*-test, *p* = 0.0094), but a greater proportion of the tobamovirus studies used older specimens than the potyvirus studies. While there is a clear trend in studies using similar time spans of samples that potyviruses have higher mean substitution rates, the conclusion based on all of the data is likely compromised by the sampling time effect. Among datasets with similar sampling times, potyviruses appear to evolve more quickly (mean rate 7.6 × 10^−4^ n/s/y) than the single representatives from four families: citrus tristeza virus, cauliflower mosaic virus, rice stunt virus and potato virus S. Potyviruses evolve at a similar rate to geminiviruses (two-tailed *t*-test, *p* = 0.15 excluding the anomalously low GRBV rate, *p* = 0.08 when including GRBV).

## 4 Discussion

Our initial screening showed CBSV had sufficient temporal signal to conduct this analysis, but UCBSV did not. CBSV has been detected longer than UCBSV (Monger et al., 2001), and previous research suggests UCBSV may be a complex of several species (Ndunguru et al., 2015) – the longer sampling times for CBSV and a more unambiguous monophyly may both contribute to CBSV being a more appropriate target for phylodynamic analysis. Clustered permutation date randomization tests conducted as a control after our analysis showed that the clustering of similar sequences sampled at the same time harmed our ability to confidently estimate the substation rate of CBSV's CP. This confounding can be due to sampling alone – it is not uncommon for plant diseases to be intensively sampled in outbreaks only, or intermittently as only a few students, separated in time, are supported to study the diversity of a pathogen (Hall et al. 2016). On the other hand, this confounding may reflect the true evolution of a pathogen, such as one that is prone to selective sweeps or the effects of genetic drift. As CBSV's diversity has declined in the observed time frame, the moderate correlation between genetic clustering and date of isolation may be either a sampling artefact or due to genuinely declining effective population size.

### 4.1 CBSV species could be a recent introduction

The coalescent analysis revealed that the most recent common ancestor of CBSV existed around 1944 (95% HPD, between years 1923–1963). Although UCBSV dataset was not included in the molecular evolutionary analysis because of its lack of temporal signal, the crude root-to-tip regression in TempEst analysis estimated that its most common ancestor existed in 1575. This datum agrees with recent reports indicating a possibility of UCBSV being older that CBSV (Ateka et al., 2017), but our estimated age of CBSV is consistent with the timing of the first observation of brown streak symptoms in cassava. Cassava was introduced into Africa by the Portuguese in the late 16th century and CBSD was first reported around Lake Malawi region in the 1930s (Storey, 1936; Nichols, 1950; Tomlinson et al., 2018). Our results support the commonly held assumption that CBSV is likely the result of an indigenous African potyvirus host-shifting into cassava (Robson et al., 2023), and it is still an emerging cassava virus (Jacobson et al., 2018; Jones, 2021; Tugume et al., 2023). This has implication on disease management as virulence and spread of newly introduced pathogens is more detrimental to sustainable food security (Jones, 2021).

### 4.2 CBSV is going through small amounts of adaptive change

Our results are similar to previous reports of limited positive selection among the CP gene of several potyviruses, with the majority of sites under purifying selection (Cuevas et al., 2012; Gao et al., 2018; Nigam et al., 2019; Wokorach et al., 2020). The study has documented evidence for pervasive purifying selection events occurring in CP gene of CBSV. The significance of the two sites under positive selection in the CP gene of CBSV needs to be established, in a manner similar to studies CP gene of potato virus Y, which identified sites associated with significant modification of viral accumulation in different hosts, as well as its transmissibility by aphids (Moury et al., 2011). Positively selected sites in the sugarcane mosaic virus CP gene were reported to have an impact on host – virus – vector interactions (Li et al., 2012). Previous mutation studies in luteoviruses have indicated that sites under purifying selection on the CP surface provide critical knowledge on the biology of those viruses (Torres et al., 2005). For example, mutations in the acidic patch domain located in the surface loop of potato leafroll virus established the role of CP in virion assembly, systematic movement and aphid transmission (Lee et al., 2005). The CP gene of vectored plant viruses, which interact with both host and vector, are under more pressure to be unchanging, as evidenced by very low ratios of the rate of non-synonymous changes to the rate of synonymous changes (dN/dS) (Chare and Holmes, 2004, Jenkins et al., 2002).

### 4.3 CBSV CP is an average evolving potyvirus CP

The substitution rate is an important parameter in understanding evolution, as high mutation rates often lead to strong adaptability and high pathogenicity in viruses (Muniraju et al., 2014; Peck and Lauring, 2018). The study has documented nucleotide substitution rates in CP gene of CBSV that are about average for a potyvirus (CBSV: 1.43 × 10^−3^ n/s/y), just slightly higher than the average of the 10 other potyviruses: 9.0 × 10^−4^ n/s/y) – but it is still a fast-evolving rate befitting of an RNA virus (Koonin et al., 2022). CP genes are often not the fastest evolving genes in viruses (Pavesi, 2021), including in CBSVs (Alicai et al., 2016), so this rate may not reflect the evolvability of other parts of the CBSV genome to overcome host resistance (Elena et al., 2014; Lafforgue et al., 2011; Montarry et al., 2012) or adaptation to altered environmental conditions or novel crops (Duffy and Holmes, 2008). Comparing across other potyviruses, turnip mosaic virus’ whole polyprotein has a higher substitution rate than that estimated for its CP (Kawakubo et al., 2021), and the P3 gene of potato virus Y has a higher substitution rate than its CP (Gao et al., 2020).

While the confounding between phylogenetic clustering and isolation date reduces the confidence we have in our substitution rate, our dataset performed robustly in other tests of temporal signal, similar to other published rates of CP evolution in plant viruses (all but eight of which assessed temporal signal by randomized dates across their sequences without regard for clustered, similar sequences). Some studies were published prior to date randomization tests being piloted (Duffy and Holmes, 2009, Ramsden et al., 2009): CABMV, MSV, RYMV, TYLCV and ZYMV. Two additional rates were calculated without BEAST (meaning no date randomization test could be run: DSV and ToSRV) and one CP rate of evolution was published without any estimate of its temporal signal (RSV, He et al. 2017). However, the whole data set randomization test has been shown to yield false negative results – that is, data sets with significant flaws in their temporal signal, such as a high correlation between clustering genetically and dates of isolation, are given potentially false credibility as having temporal signal (Duchene et al. 2015, Murry et al. 2016). Therefore, we employed a more modern approach to verifying the temporal signal in our dataset and received strong support (Duchene et al. 2020). This support did not hold up to clustered permutation date randomization, implying that our calculated substitution rate should be interpreted with caution as it may be inaccurate. Arguably most of the collated plant virus rates have similarly confounded datasets, because of the common problem of uneven sampling practices (Hall et al. 2016; Murray et al. 2016). Therefore, our comparisons among rates are apples-to-apples – with potentially many of these rates needing revisiting in the future, when hopefully there will be more unbiased sampling of the viruses over time, and improvements in methods for pruning datasets to reduce the confounding effects of phylogenetic clustering and dates of isolation.

The differences of individual datasets, and the idiosyncratic ecological conditions of each virus, lead to variation in substitution rates even if the viruses within each family have similar mutation rates, complicating detection of overarching patterns (Hicks and Duffy, 2014). Nonetheless, potyviruses appear to evolve more quickly than several other plant virus families based on the limited data available. Potyviruses are known to be capable of significant diversity (Nigam et al., 2019) and have high mutation frequencies (Khanal and Ali, 2021). Their more frequent emergence on novel hosts (Gibbs et al., 2020; Moury and Desbiez, 2020) may be due to higher rates of evolution than some other families of plant viruses.

## 5 Conclusion

CBSV, but not UCBSV, has been sufficiently sequenced in number and over sufficient time in East Africa to support a tip-dated coalescent analysis of its rate of evolution, which is just as expected for a member of *Potyviridae*.

## Author statement

No AI technologies (large language models or otherwise) were used in the analysis or preparation of this manuscript.

## CRediT authorship contribution statement

**Willard Mbewe:** Writing – review & editing, Writing – original draft, Methodology, Formal analysis, Data curation, Conceptualization. **Settumba Mukasa:** Writing – review & editing, Supervision. **Mildred Ochwo-Ssemakula:** Writing – review & editing, Supervision, Resources. **Peter Sseruwagi:** Writing – review & editing, Supervision. **Fred Tairo:** Writing – review & editing, Supervision, Funding acquisition. **Joseph Ndunguru:** Writing – review & editing, Supervision, Funding acquisition. **Siobain Duffy:** Writing – review & editing, Visualization, Resources, Methodology, Formal analysis, Conceptualization.

## Declaration of competing interest

The authors declare that they have no known competing financial interests or personal relationships that could have appeared to influence the work reported in this paper.

## Data Availability

All data used is publicly available on GenBank or in the published literature.

## Appendix Supplementary materials

Image, application 1

Image, application 2
