# Supplementary material for: Cassava brown streak virus evolves with a nucleotide-substitution rate that is typical for the family Potyviridae
Source: Virus Res. 2024 May 22;346:199397. doi: 10.1016/j.virusres.2024.199397 (PMC11145536; doi:10.1016/j.virusres.2024.199397)
Supplement: Supplementary file 2 [file mmc2.pdf]

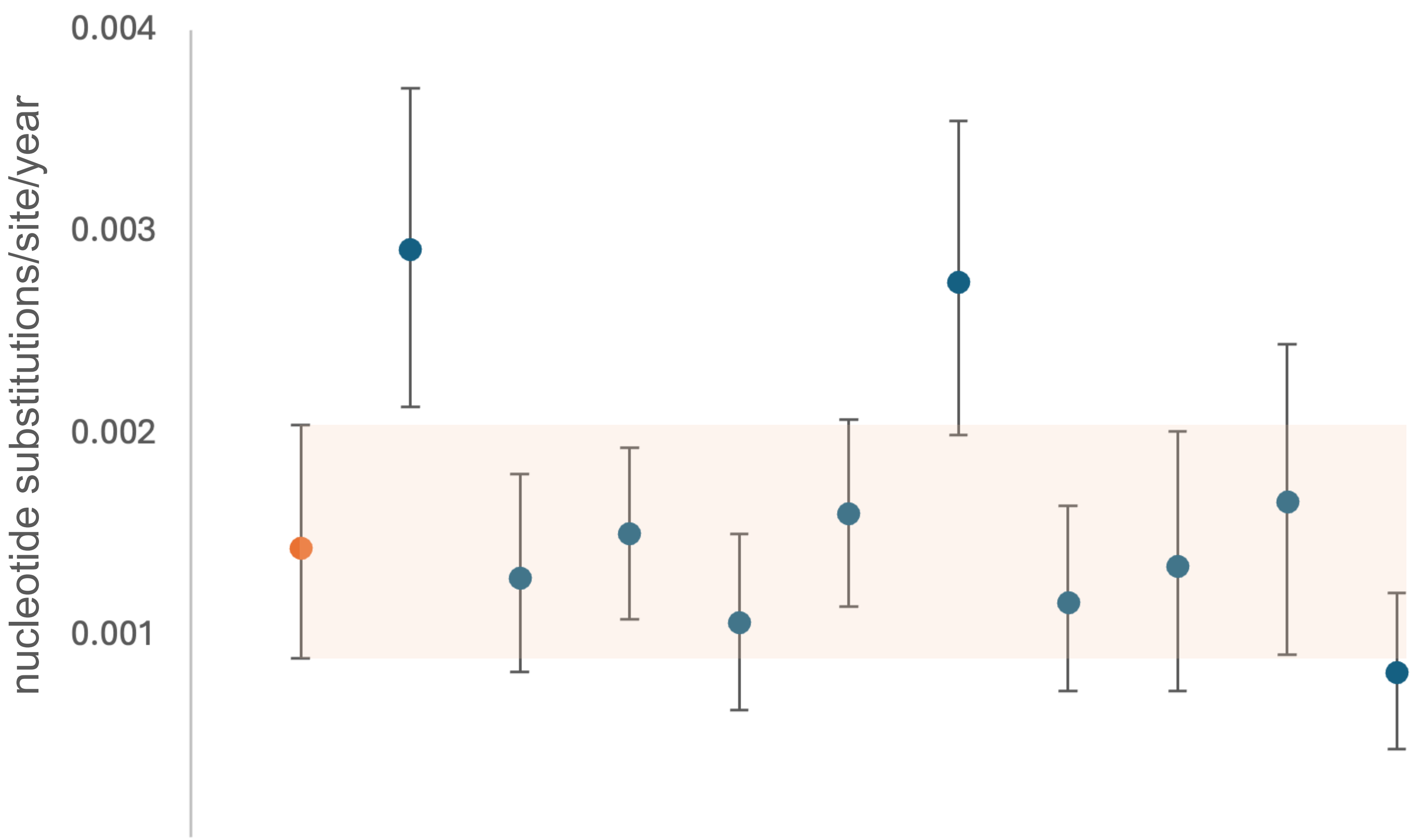

Suppl Figure 2: the CBSV substitution rate (with 95% credibility interval) plotted in orange and those of 10 cluster permutated date randomized data sets with the same priors. The 95% credibility interval is shaded in orange across the 10 other rates, most randomized estimates fall within this range.
